# Clinician Participation in Innovation Labs at University Hospitals: Mixed Methods Study

**DOI:** 10.2196/84887

**Published:** 2026-08-07

**Authors:** Louis Agha-Mir-Salim, Thorsten Adami, Anne Rike Flint, Anette Ströh, Felix Balzer, Akira-Sebastian Poncette

**Affiliations:** 1 Institute of Medical Informatics Charité – Universitätsmedizin Berlin, Corporate Member of Freie Universität Berlin and Humboldt-Universität zu Berlin Berlin Germany; 2 Einstein Center Digital Future Berlin, Berlin Germany; 3 Charité BIH ARC Innovation Center Charité – Universitätsmedizin Berlin, Corporate Member of Freie Universität Berlin and Humboldt-Universität zu Berlin Berlin Germany

**Keywords:** change management, digital transformation, health care innovation, hospital staff, innovation lab, innovation, mixed methods, staff engagement

## Abstract

**Background:**

Innovation labs (ILs) are increasingly being implemented in hospital settings to propel collaboration and experimentation against the backdrop of digital transformation. While these labs offer substantial potential for accelerating the development and testing of novel clinical and operational solutions, the sustainable participation of hospital staff remains a challenge. Frontline clinicians and nurses possess essential contextual knowledge yet face significant structural, cultural, and time-related barriers to meaningful IL engagement. Understanding the conditions under which hospital staff can be effectively integrated into ILs is therefore critical to realizing their transformative potential.

**Objective:**

This study aims to explore how hospital staff can be effectively engaged in ILs within a university hospital setting. The specific objectives are to (1) identify organizational, structural, and cultural conditions that enable or hinder participation; (2) understand enablers and barriers from the perspectives of staff across professional groups; (3) characterize differences between physician and nursing perspectives on IL engagement; and (4) derive practical recommendations for the design and implementation of ILs.

**Methods:**

An exploratory sequential mixed methods design was used for this study, conducted at Charité – Universitätsmedizin Berlin, a large university hospital in Germany. In phase 1, semistructured interviews were conducted with innovation leaders from nonhospital sectors (group A) and clinical and nonclinical leaders from the study hospital (group B). Themes derived from the qualitative analysis informed the development of a structured online survey. In phase 2, this survey was distributed to all hospital staff across campuses. Quantitative data were analyzed using descriptive and nonparametric comparative methods (Mann-Whitney *U* test) to assess differences between professional groups.

**Results:**

In the qualitative phase, 13 interviews identified 8 emergent themes across 3 typological categories: strategic alignment of ILs, design principles, and staff motivation. In the quantitative phase, 532 responses were collected via the structured 44-item online survey (226/334, 67.7% physicians; 53/334, 15.9% nurses). Findings reveal strong staff support for ILs that align with institutional priorities (eg, patient care and digital transformation), prioritize workflow improvements, and are embedded within hospital structures. Key enablers include protected time, dedicated funding, and access to methodological expertise. Participants were motivated by opportunities to acquire new skills and address clinical problems, preferring flexible roles over commitments extending beyond clinical hours or participation in spin-offs. Statistically significant differences between professional groups were observed in motivation patterns, perceptions of innovation outcomes, and structural needs.

**Conclusions:**

Staff perspectives indicate that engaging clinicians in university hospital-based ILs requires structural support, visible strategic alignment, and attention to differing professional motivations. Internal IL models, resource allocation, and tailored participation formats appear essential for clinician engagement. While investment is required, this approach may yield substantial returns through enhanced care delivery, staff satisfaction, and organizational adaptability. We present empirically grounded, actionable recommendations for IL design and implementation in university hospitals.

## Introduction

Innovation has become a strategic imperative across industries, enabling organizations to streamline workflows and integrate emerging technologies into routine operations [1]. Health care is no exception: faced with persistent inefficiencies, resource constraints, and the urgent need for digital transformation, health systems increasingly rely on innovation to improve care delivery and organizational performance [2-4]. In this context, health care innovation is understood as any change that enables practitioners to deliver patient care more efficiently, effectively, and economically [5].

Health care innovation is fundamentally need driven, emerging in response to identified clinical or operational gaps rather than spontaneous invention [6,7]. To systematically identify and address such needs, health care organizations are increasingly establishing innovation labs (ILs): dedicated physical, virtual, or hybrid environments designed to promote collaboration, experimentation, and problem-solving [2]. ILs function as structured incubators for prototyping and testing novel solutions, guiding the adoption of emerging technologies, and accelerating the translation of clinical needs into operational practice [4]. They bring together multidisciplinary stakeholders, including clinicians, designers, engineers, researchers, and business experts, to co-develop approaches aimed at enhancing patient and provider experiences and strengthening health system performance [8,9]. The term IL is used variably across the literature. For the purposes of this study, we define an IL as a permanent or semipermanent organizational unit embedded within a hospital that provides the infrastructure, resources, and processes necessary for the systematic development and testing of novel clinical and operational solutions. Within this structure, we define an IL project as a discrete, time-limited initiative with a clearly defined problem, team, and deliverable, conducted within or supported by an IL.

However, the success of innovation initiatives depends heavily on the meaningful, rather than nominal, participation of frontline clinical staff, who provide critical contextual knowledge and are often the primary users of the solutions developed [10]. Despite this recognized value, health care professionals remain systematically underrepresented in innovation efforts: studies have documented low clinician participation rates in initiatives such as health care hackathons [11,12], as well as shortcomings in the integration of clinical domain expertise in technically oriented research targeting health care challenges [13,14]. More broadly, implementing health care innovations faces resistance from professionals and organizations alike, compounded by resource limitations, communication barriers, and insufficient stakeholder involvement [15]. These patterns reflect structural barriers, including time constraints, limited institutional support, and underdeveloped innovation cultures, that frequently impede meaningful engagement [8,10]. Individual-level factors, such as resistance to change or limited capacity to participate in innovation activities, can further compound these constraints [16]. Addressing these barriers is therefore regarded as a prerequisite for realizing the potential of ILs in hospital settings.

While existing literature highlights the growing role of ILs in health care transformation [2,4,8,9,17,18], the conditions under which these barriers can be overcome remain poorly understood, particularly with regard to how hospital staff, especially clinicians, can be effectively engaged in such environments. University hospitals occupy a distinctive position in this regard: their research mandates, academic structures, and dual clinical-academic staff roles make them natural adopters of ILs as part of broader digital transformation strategies [4], yet also expose them to particular engagement challenges. There is an urgent need to identify and address the organizational, structural, and individual factors that influence staff participation in hospital-based ILs. To address this gap, we used an exploratory sequential mixed methods design combining qualitative perspectives from cross-sector innovation leaders and hospital-based leadership with a large-scale quantitative staff survey, an approach not previously applied in the health care IL literature.

The aim of this study is to explore how hospital staff can be meaningfully engaged in ILs within a university hospital setting. To achieve this, the study pursues four interrelated objectives: (1) to gather qualitative insights from innovation leaders from nonhospital settings to identify potentially transferable strategies for the development of ILs in hospitals; (2) to identify structural, organizational, and cultural enablers and barriers to IL participation from the perspectives of clinical and nonclinical hospital leadership; (3) to quantitatively assess the perceived barriers and facilitators among hospital staff within a university hospital; and (4) to develop practical, empirically derived recommendations for the design and implementation of ILs in university hospitals.

## Methods

### Ethical Considerations

The study was approved by the institutional ethics committee of Charité – Universitätsmedizin Berlin (EA4/039/22). All participants received written information about the study and provided informed consent prior to participation. Data collection, storage, and processing complied with institutional policies and the General Data Protection Regulation. Compensation was not provided to participants.

### Study Design and Setting

This study used an exploratory sequential mixed methods design and was conducted at a large university hospital in Germany. In phase 1, qualitative data were collected through semistructured interviews to explore key themes related to the development and implementation of ILs. Insights from this phase informed the design of a structured online survey administered in phase 2 to assess the broader relevance and prevalence of these themes across hospital staff. The 2 phases therefore intentionally involved different samples serving complementary roles: phase 1 purposively targeted leadership perspectives to generate all relevant themes for IL conceptualization and implementation, while phase 2 surveyed the staff population as the primary stakeholders of a hospital IL. Findings from both phases were integrated to develop practical recommendations for implementing ILs in university hospital settings. We report in accordance with the SRQR (Standards for Reporting Qualitative Research) [19] for the qualitative phase and the CROSS (Checklist for Reporting of Survey Studies) [20] for the quantitative phase; completed checklists are provided in Multimedia Appendix 1.

### Participant Recruitment and Sampling

#### Qualitative Phase

Purposive sampling was used to recruit participants from two groups: (1) innovation experts from cross-sector, nonhospital settings and (2) clinical and nonclinical leaders from the university hospital. Group A was included to broaden the initial theme space and identify transferable organizational and structural strategies from outside health care, thereby preventing the interview guide and emergent coding from being overly constrained by hospital-specific assumptions. This cross-sector perspective was intentional given the absence of comparable IL structures at the scale of German university hospitals, making external expertise the most viable reference point for transferable strategies. Group A participants were required to hold a current senior or management role at an IL focused on the development of internal digital innovation or the translation of digital products or services into operational practice. Sectors were explicitly diversified, spanning pharmaceuticals, aviation, financial services, insurance, defense, real estate, technology, and municipal government, to maximize transferability across organizational contexts. ILs focused primarily on external venture creation, startup incubation, or spin-off activities without a direct implementation focus were excluded. A total of 12 individuals were approached through online searches and professional networks (LinkedIn) via direct outreach. As purposive qualitative sampling does not require a formal sample size calculation, none was conducted; instead, thematic saturation guided sample adequacy. Group B included clinical leaders and innovation unit leads at Charité – Universitätsmedizin Berlin, reflecting the governance reality of academic medical centers in which IL implementation spans both clinical and administrative functions. A total of 6 eligible individuals were approached via institutional email. No other formal exclusion criteria were applied.

#### Quantitative Phase

Following qualitative analysis, a structured online survey was distributed to hospital staff across all Charité campuses. While all hospital staff were considered eligible to participate, recruitment efforts focused on clinical professions as the primary stakeholders of ILs. Physicians were recruited via a Charité-wide physician mailing list. As no equivalent mailing list existed for nursing staff, the survey was distributed to all nursing team leads across Charité wards, who were invited to forward the invitation to their teams. Participation was voluntary and anonymous. As is standard for exploratory descriptive surveys, no formal sample size calculation was conducted.

### Data Collection

#### Qualitative Phase

Interviews were conducted in German using a semistructured interview guide and were subsequently audio-recorded, transcribed verbatim, and anonymized. They were conducted between February and September 2023 via Microsoft Teams video conferencing. An English translation of the interview guide is available in Multimedia Appendix 2.

#### Quantitative Phase

Data collection targeted all hospital staff as potential stakeholders in ILs, with a focus on health care professionals. Eligible participants were invited via email, which included study information and a link to the online survey. Data collection took place between September 2024 and February 2025, with a reminder email sent 8 weeks after the initial invitation to encourage participation. Data were collected using REDCap (version 14.5.25) [21].

### Data Analysis

#### Qualitative Phase

Data were coded using the method of typological analysis [22], consisting of three steps: (1) identification of attributes based on inductive coding of transcripts, (2) organization of attributes into themes, and (3) synthesis of themes into overarching typological categories. Coding disagreements and theme development were discussed among the research team. Thematic saturation was informally assessed by the research team during analysis for each group separately; no new themes emerged in the final 2 to 3 interviews of each group, suggesting adequate coverage for the purpose of survey item development. Coding and data management were conducted using MAXQDA software (version 24.2.0; VERBI GmbH).

#### Data Integration and Survey Development

Qualitative and quantitative data were integrated during both survey design and interpretation. Qualitative findings provided the conceptual basis for item development and survey structure. Quantitative results were later compared with qualitative themes to assess consistency and identify agreements or divergences.

The survey included demographic items (gender, years of professional experience, primary responsibilities, and profession) and 44 closed-ended items organized under inductively derived categories and themes. Except for demographic items, all items used 5-point Likert scales. Items were developed iteratively by the research team, drawing on interview material and theme summaries. Focus groups were conducted to assess content relevance and coverage; cognitive interviews were subsequently used to evaluate item-level clarity and face validity. Prior to full deployment, the instrument was piloted with staff from the hospital’s IT department to assess comprehensibility, estimate completion time, identify ambiguous items, and verify technical functionality. To reduce respondent burden, no items were mandatory. The survey was administered in German, with an English translation available in Multimedia Appendix 3. As the instrument was purpose built from qualitative findings rather than adapted from an existing validated scale, psychometric evaluation was outside the scope of this study.

#### Quantitative Phase

Descriptive statistics were calculated, including medians, IQRs, and 95% CIs. As survey items were nonmandatory, each item was analyzed based on all available responses; denominators therefore vary across items and reflect the number of respondents who answered that particular item. For the comparative analysis of the main clinical stakeholders of interest (physicians and nurses), the Mann-Whitney *U* test was applied. To control the false discovery rate across multiple item-level comparisons, raw *P* values were adjusted using the Benjamini-Hochberg procedure, with significance evaluated at a false discovery rate of 5%.

All quantitative analyses were performed using Python (version 3.13.1) in a Jupyter Notebook (version 7.3.2). The following Python packages were used: *Pycap* (version 2.6.0), *Pandas* (version 2.2.3), *NumPy* (version 2.2.1), *SciPy* (version 1.14.1), *Matplotlib* (version 3.10.0), *Seaborn* (version 0.13.2), and *Plot-Likert* (version 0.5.0).

### Reflexivity

This study was conducted by a multidisciplinary team with backgrounds spanning medicine, business administration, design, and medical informatics, based at the study hospital (Charité – Universitätsmedizin Berlin). Several team members work within or adjacent to the institutional innovation ecosystem, which may have shaped both the framing of the research questions and the interpretation of the findings, particularly regarding the feasibility and desirability of ILs as an organizational model. We attempted to mitigate this through the inclusion of cross-sector external perspectives in phase 1, an iterative team-based analytic process, and transparent reporting of methods and sampling decisions. Nonetheless, readers should consider that the research team’s institutional position may introduce a degree of confirmation bias toward viewing ILs favorably as instruments of hospital transformation.

## Results

### Qualitative Phase

#### Participants

A total of 13 participants took part in the qualitative phase, of whom 7 were affiliated with existing external ILs and 6 held hospital leadership roles. The professional backgrounds and positions of the participants are presented in Table 1.

**Table 1:** Participant professional backgrounds of the qualitative phase.

Group	Position	Industry	Participants, n
A	Managing director	Municipal government	1
A	Head of department	Pharmaceuticals industry, financial services	2
A	Director	Insurance	1
A	Product manager	Defense, real estate financing	2
A	Analyst	Aviation	1
B	Medical department director	Health care (study hospital)	2
B	Head of hospital innovation unit	Health care (study hospital)	2
B	Nursing team leads	Health care (study hospital)	2

#### Overview of Themes

Across all 13 interview transcripts, 58% of the total content was assigned at least 1 code during typological analysis, yielding 41 unique attributes. Transcript segments not assigned a code consisted of contextual narrative and preamble that fell outside the scope of the 3 typological categories. The 41 attributes were clustered into eight overarching themes: (I) key challenges, (II) IL outcomes, (III) IL strategy, (IV) IL design principles, (V) cultural aspects, (VI) roles and skills, (VII) organizational integration, and (VIII) staff motivation.

#### Theme 1: Key Challenges

Establishing and operating an IL presents several key challenges that must be addressed in both its design and execution. A primary concern is integration: “The biggest challenge for an innovation lab is integration into the organization” (Int_Interview1).

When labs operate too independently, their work may lack strategic alignment: “If labs operate too far from the organization, castles in the sky emerge—the strategic fit is missing” (Ext_Interview1).

Other challenges include the tension between innovative, agile approaches and established institutional processes, the slow pace of change, and the absence of institutionalized collaboration mechanisms. Difficulties often arise during the transition from experimentation to implementation: “Handover of the MVP into the line is a breaking point—ownership and sustainable support are often lacking” (Ext_Interview7).

Furthermore, pulling highly motivated individuals into the IL can leave core functions understaffed. There is also the risk that innovation outputs fail to create lasting impact. Despite these barriers, the urgency to innovate is increasing:

The time for innovation is now, because business as usual is no longer enough—staff shortages, digitization, demographic change etc. require new approaches.

Int_Interview6

Another key barrier to success can arise from limited collaboration with other parts of the institution, which can further hinder the lab’s ability to scale or embed innovations sustainably.

#### Theme 2: IL Outcomes

IL projects generate outcomes that extend beyond the lab itself, for example, introducing new approaches, transferring knowledge, and creating value for the wider organization. These outcomes can be sparked by different inputs, such as external ideas, requests from business units, or internally driven initiatives. In this way, ILs act as multipliers, turning diverse inputs into impactful organizational results: “Our labs generate multipliers: former product owners later build up digital structures” (Ext_Interview6).

These efforts result in a range of tangible outcomes: “In the end, there are always tangible products: MVPs, apps, new services that are measured” (Ext_Interview7).

However, beyond products, ILs also contribute to a broader transformation of care models and mindsets:

In the end, it's about rethinking care—patients don’t always have to come to the university hospital in person.

Int_Interview4

#### Theme 3: IL Strategy

A clear strategy defines the direction of an IL and determines how it contributes to the broader organizational goals. Strategic considerations include whether to focus on incremental improvements or disruptive innovation, how to position the IL within the organization, and the extent to which external startups or products are integrated. The strategy also shapes how the IL supports digital transformation, connects internal and external actors, and empowers entrepreneurial thinking.

To be effective, IL projects need alignment with broader business objectives and visible executive support: “Projects work best when they have both a business impact and strategic commitment” (Ext_Interview6).

Anchoring the IL within strategic units of the organization is seen as essential for long-term relevance: “An IL needs strategic anchoring, eg, in the division of strategic development” (Int_Interview6).

A shift in mindset is also part of the strategic agenda: “We must move out of the saving mentality and into a willingness to innovate, with the right mindset” (Int_Interview4).

#### Theme 4: IL Design Principles

Design principles provide the operational framework for an IL, including its governance structure, legal setup, budget, and physical infrastructure. These principles define how the lab functions day to day and how it aligns with the broader organization: “We have our own budget, which we can manage independently. This gives us strategic freedom” (Ext_Interview2).

A well-defined operating model ensures clarity in roles, processes, and decision-making structures. Interdisciplinary collaboration is often embedded in the lab’s design: “We always had highly interdisciplinary teams: from design, IT, product development to data science” (Ext_Interview1).

Contextual and domain-specific proximity to actual care delivery is also emphasized: “An IL must be close to real care processes, not in an ivory tower” (Int_Interview4).

#### Theme 5: Cultural Aspects

The culture within an IL plays a central role in shaping how new ways of working, such as design thinking or agile methods, are adopted and disseminated. A strong cultural foundation supports experimentation, learning from failure, and collaboration while also influencing broader organizational mindsets when IL staff return to their line functions: “Innovation culture cannot be dictated. It must be experienced and role-modeled” (Ext_Interview3).

A psychologically safe space is essential to enable risk-taking and open dialogue: “A platform for openly discussing mistakes, failure, and learning is needed. Otherwise no one dares to innovate” (Int_Interview1).

Genuine participation, rather than symbolic engagement, helps build credibility and fosters cultural change: “We must move away from the ivory tower. Real involvement is crucial for acceptance” (Int_Interview4).

#### Theme 6: Roles and Skills

Effectively integrating the right stakeholders along with their relevant knowledge, skills, and attitudes is essential for successfully executing IL projects: “Product owner from line function brings in subject matter expertise and ownership” (Ext_Interview7).

Assembling diverse teams ensures a broad range of skill sets aligned with the specific needs of IL projects: “IL teams consist of UX, development, product, data science—classic roles, differently organized” (Ext_Interview2).

Beyond technical capabilities, having the right mindset within the team is also critical:

Nurses: Younger staff are often more motivated.

Int_Interview3

Young senior physicians or specialists are ideal—motivated, still flexible, with a view into clinical life.

Int_Interview1

#### Theme 7: Organizational Integration

Ensuring strong collaboration and clear interfaces between the IL and clinical departments, as well as other parts of the university hospital, is seen as vital: “Labs that plan reintegration work better, otherwise the outcome has no home” (Ext_Interview6).

To ensure accountability and continuity, it is important to initiate handover processes early: “Handover works best through co-development—otherwise it fails due to lack of responsibility” (Ext_Interview2).

Clearly defined transition points between the IL and operational departments are necessary for lasting success: “A clear handover point is missing—without IT and line integration, many projects fizzle out” (Int_Interview6).

#### Theme 8: Staff Motivation

Motivating staff is a key factor in attracting talent and driving meaningful outcomes in IL projects. New ways of working can act as a strong motivational driver:

Access to a different working world: new methods, new team formats, new environments create appeal.

Ext_Interview3

Experiencing new ways of working makes people rarely want to go back—it attracts them back to the IL.

Int_Interview3

Working on meaningful and relevant topics also serves as a strong intrinsic motivator: “The biggest motivator is the topic itself—not bonuses or titles” (Ext_Interview2).

Seeing the tangible impact reinforces motivation: “Motivation arises from experiencing one's own impact” (Int_Interview6).

Allowing space for personal initiative keeps engagement high: “If you can implement ideas, you stay engaged—otherwise frustration builds” (Int_Interview4).

### Integration of Qualitative and Quantitative Findings

The 8 themes were synthesized and grouped to form 3 main typological categories:

- Strategic alignment of ILs, focusing on the institutional positioning of ILs;
- Design principles of ILs, describing necessary frameworks and conditions;
- Motivating employees to participate, addressing individual drivers and expectations.

The final survey included 15 items for category I, 11 items for category II, and 18 items for category III.

**Figure 1:**
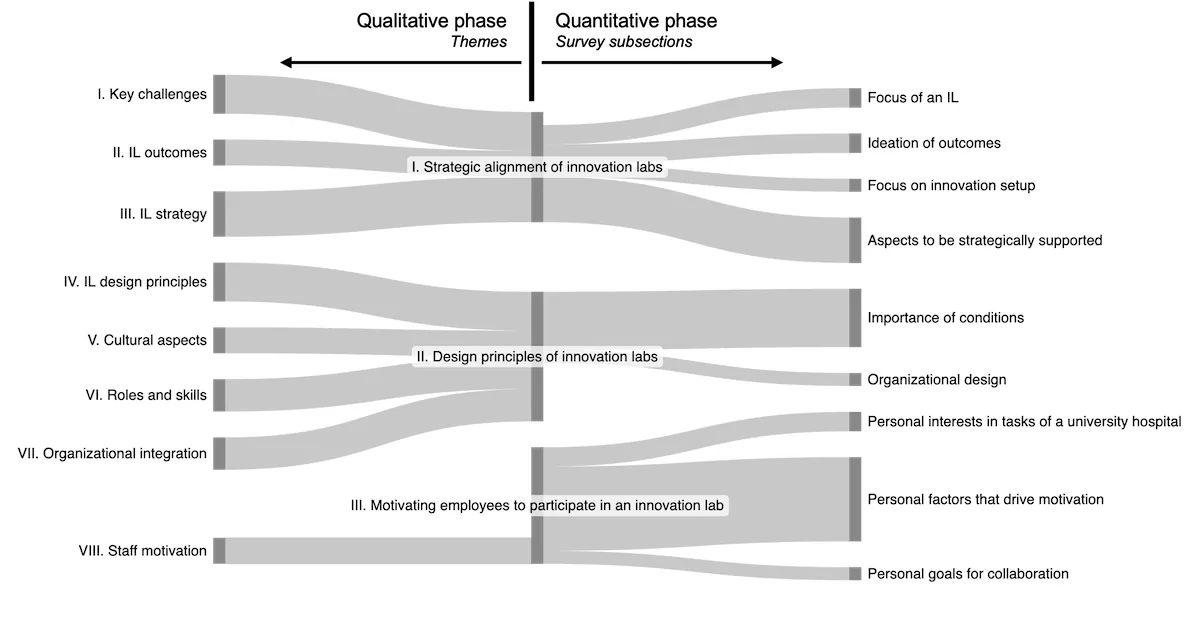
The 41 attributes were grouped into 8 themes (left), which were then combined into 3 typological categories (middle). These categories shaped the 44-item questionnaire, where each survey item was organized by category and further divided into thematic subsections (right). IL: innovation lab.

The items in each category were organized into thematic subsections. Figure 1 illustrates the structure of this organization. It shows how the themes, under which the initial attributes were grouped, were assigned to the categories, which informed the structure and subsections of the survey.

### Quantitative Phase

#### Participants

**Figure 2:**
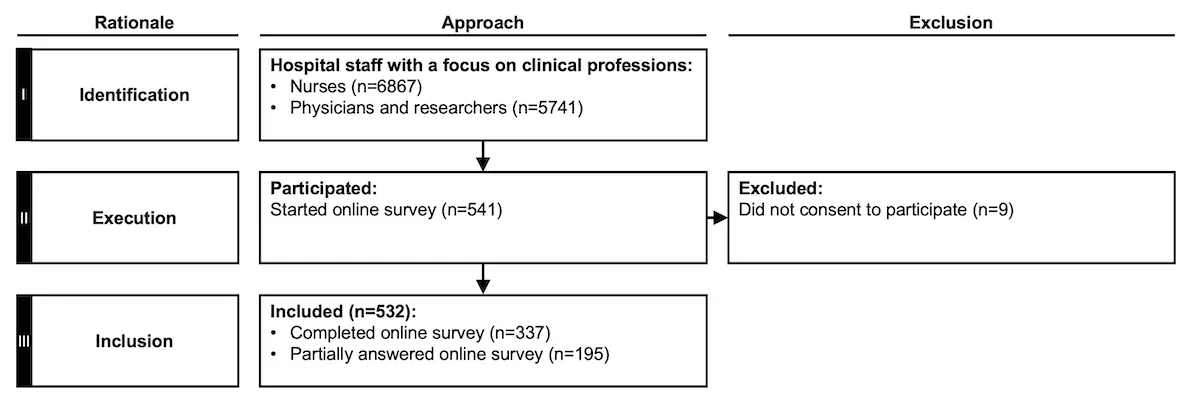
Participant flowchart.

An online survey was distributed among staff members at a German university hospital, yielding a total of 532 valid responses, including 337 complete responses. The exact number of individuals reached could not be determined; therefore, a formal response rate could not be calculated. A flowchart of participant inclusion is presented in Figure 2. Participant demographics are presented in Table 2.

**Table 2:** Participant demographics from the quantitative phase. Responses to all fields were optional; therefore, totals may not sum to the total number of responses (N=532).

Category and subcategory			Participants, n (%)
Gender			
	Female	182 (55)	
	Male	141 (42.6)	
	Nonbinary	1 (0.3)	
	Prefer not to say	7 (2.1)	
Profession			
	Physician	226 (67.7)	
	Nurse	53 (15.9)	
	Trainee/student (medical/nursing)	11 (3.3)	
	Other health care professional	6 (1.8)	
	Other	38 (11.4)	
Professional experience (years)			
	0-5	86 (25.7)	
	6-10	59 (17.6)	
	>10	190 (56.7)	
Dominant field of work^a^			
	Patient care	258 (48.5)	
	Research	159 (29.9)	
	Education	118 (22.2)	

^a^Participants could select multiple categories, so percentages sum to more than 100%.

#### Survey Findings

The following sections present detailed survey results, structured according to the 3 typological categories identified in the study. All survey results are presented in Multimedia Appendix 4. Due to the overrepresentation of physicians in the sample (226/334, 67.7% vs 53/334, 15.9% nurses), comparisons between groups should be considered exploratory and interpreted as such.

A further consideration in interpreting the findings is the study site’s innovation landscape. The BIH Digital Health Accelerator supports Charité clinicians and researchers through structured funding calls, entrepreneurship training, and mentorship programs. Charité’s “Healthcare Transformation Workshop,” launched in 2024, provides a framework for implementing clinician-initiated improvements of operational value to the hospital; since 2025, it has co-organized a hospital-wide ideas contest (“What-if-a-thon”) to foster bottom-up innovation. The local “Ward2030 program”, in operation since 2022, applies design thinking methods to empower interdisciplinary clinical teams to redesign ward-level workflows. Staff familiarity with these initiatives and the relatively mature innovation culture at Charité may have influenced survey responses, potentially inflating endorsement of IL-oriented items compared with institutions at earlier stages of innovation maturity.

#### Category I: Strategic Alignment of ILs

##### Overview

This category addresses the institutional positioning of ILs within a university hospital. The corresponding survey items were organized into 4 subsections. The first focuses on how an IL’s purpose should align with the hospital’s core missions of research, education, and patient care. The second examines the outcomes that ILs should prioritize. The third explores the strategic setup of ILs within the broader organizational context. The fourth subsection identifies areas where ILs can provide targeted support to further strengthen the university hospital’s overall strategy.

##### Focus of an IL (S1-S3)

Participants broadly endorsed the role of ILs in supporting the 3 core missions of a university hospital (Multimedia Appendix 5). The highest level of agreement was reported for patient care (S1: 394/431, 91.42% agreed or strongly agreed), followed by research (S3: 362/413, 87.65%) and education (S2: 326/409, 79.71%). These results suggest strong overall alignment between IL activities and the hospital’s strategic objectives across domains. No statistically significant differences were observed between physicians and nursing staff for any of these items.

##### Ideation of Outcomes (S4-S6)

**Figure 3:**
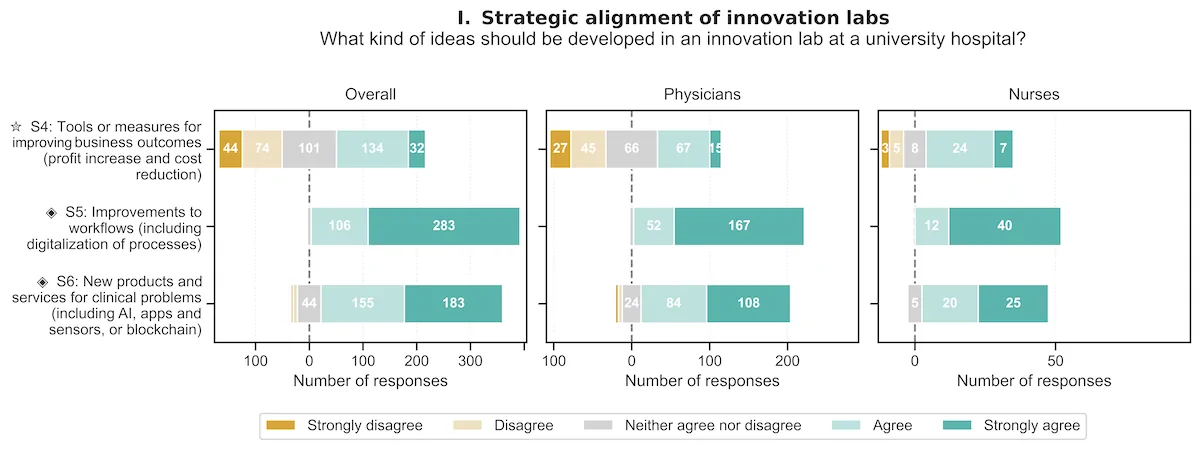
Overall responses to strategic alignment questions S4 to S6, focusing on the ideation of outcomes for an innovation lab within a university hospital. Responses marked with diamonds have a median ≥4, and stars indicate statistically significant differences between professional groups.

In terms of outcomes, respondents expressed the highest support for IL activities aimed at improving workflows, including the digitalization of processes (S5: 389/397, 97.98% agreed or strongly agreed, Figure 3). Similarly, a strong majority endorsed the development of new products and services to address clinical problems (S6: 338/394, 85.79%). In contrast, there was notably lower agreement for initiatives focused on improving the business model, such as increasing grant income or reducing costs (S4), with 30.65% (118/385) of participants expressing disagreement or strong disagreement. This item also revealed significant differences between professional groups (S4: *U*=3608.5; *P*=.007), indicating divergent views on the value of financially oriented innovation across roles, with higher agreement among nurses.

##### Focus on Innovation Setup (S7-S8)

Regarding the structural focus of innovation activities, 92.49% (357/386; S7) of respondents agreed or strongly agreed with internal innovation within the university hospital, while 73.75% (281/381; S8; Multimedia Appendix 6) endorsed external collaboration, such as with startups. No statistically significant differences were observed between professional groups for either item.

##### Aspects to Be Strategically Supported (S9-S15)

**Figure 4:**
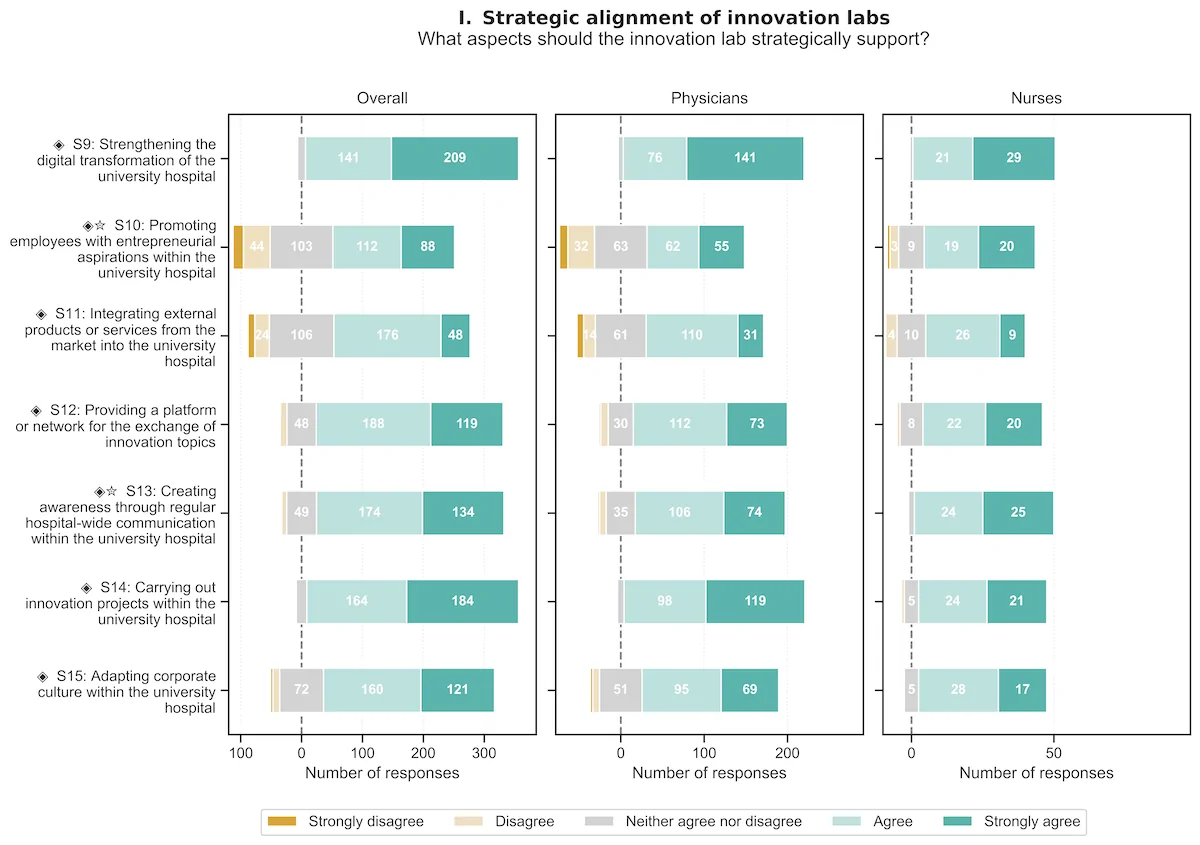
Overall responses to strategic alignment questions S9 to S15, focusing on aspects to be strategically supported within a university hospital. Responses marked with diamonds have a median ≥4, and stars indicate statistically significant differences between professional groups.

Survey responses revealed strong support for those aspects of IL activities aligned with digital transformation goals and the execution of IL projects (Figure 4). Specifically, 95.89% (350/365; S9) of participants agreed or strongly agreed that ILs should support digital transformation within the university hospital, while 94.82% (348/367; S14) agreed or strongly agreed with the strategic value of carrying out IL projects. Similarly high levels of agreement were observed for raising awareness through regular hospital-wide communication (S13: 308/367, 83.92%) and for establishing platforms or networks to facilitate the exchange of innovation-related topics (S12: 307/368, 83.42%). Support for adapting the corporate culture to foster innovation was slightly lower but still substantial, with 76.36% (281/368; S15) of respondents agreeing or strongly agreeing.

More varied responses were observed for integrating external products or services into the hospital context (S11: 224/365, 61.37%) and for promoting entrepreneurial aspirations among staff (S10: 200/364, 54.95%), the latter of which also had the highest rate of disagreement (61/364, 16.76% disagreed or strongly disagreed). Notably, significant differences between professional groups were identified for items S10 (*U*=4325.5; *P*=.02) and S13 (*U*=4368; *P*=.02), with higher agreement among nurses.

#### Category II: Design Principles of ILs

##### Overview

This category explores how ILs should be structured and operated within the hospital context. The survey items are divided into 2 subsections. The first focuses on the conditions considered important for successful collaboration. The second captures participants’ views on the organizational design of ILs.

##### Importance of Conditions (D1-D9)

Respondents rated several conditions as important prerequisites for participation in ILs (Multimedia Appendix 7). The highest levels of agreement were reported for working time allocation (D1: 336/353, 95.18% agreed or strongly agreed), sufficient funding (D2: 337/353, 95.47%), and access to a dedicated space for collaboration (D3: 329/350, 94%). Other frequently endorsed conditions included access to necessary skills or experts (D4: 324/354, 91.53%), clearly defined organization and processes (D5: 293/347, 84.44%), legal clarity (D6: 282/351, 80.34%), and linkage with business areas of the university hospital (D7: 268/352, 76.14%). Modern collaboration methods, such as agile working and design thinking (D8: 260/354, 73.45%), and financial compensation (D9: 243/352, 69.03%) received comparatively lower, yet still substantial, levels of support.

Statistically significant differences between physicians and nurses were observed for 2 items: linkage with business areas (D7: *U*=3314; *P*<.001) and use of modern methods of collaboration (D8: *U*=4089.5; *P*=.007), with higher agreement among nurses (Multimedia Appendices 4 and 7).

##### Organizational Design (D10-D11)

**Figure 5:**
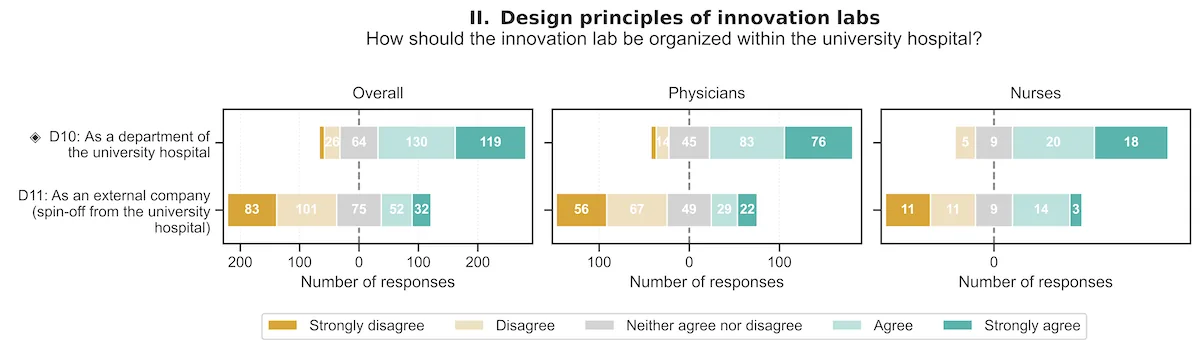
Overall responses to design principles questions D10 to D11, focusing on the organizational design of an innovation lab within a university hospital. Responses marked with diamonds have a median ≥4, and stars indicate statistically significant differences between professional groups.

Regarding the preferred organizational setup, 71.55% (249/348; D10) of participants agreed or strongly agreed with an internal IL structure integrated within the university hospital (Figure 5). In contrast, only 24.49% (84/343; D11) favored the establishment of an external IL as a spin-off company, while 53.64% (184/343) explicitly disagreed or strongly disagreed with this external model. No statistically significant differences were observed between professional groups for either item.

#### Category III: Motivating Employees to Participate in an Innovation Lab

##### Overview

This category focuses on factors that encourage staff to engage in innovation activities. The survey items are organized into 3 subsections. The first explores personal interests related to the core tasks of a university hospital. The second examines individual motivation factors. The third captures personal goals for participating in collaborative work within an IL.

##### Personal Interests in Tasks of a University Hospital (M1-M3)

When asked which university hospital tasks would most motivate their participation in ILs, 88.37% (304/344; M1) of respondents selected patient care, followed by research at 67.54% (233/345; M3) and education at 59.23% (199/336; M2; Multimedia Appendix 8). A statistically significant difference between professional groups was found for research (M3: U=7519; *P*<.001), with higher agreement among physicians, while no significant differences were observed for patient care or education.

##### Personal Factors That Drive Motivation (M4-M16)

**Figure 6:**
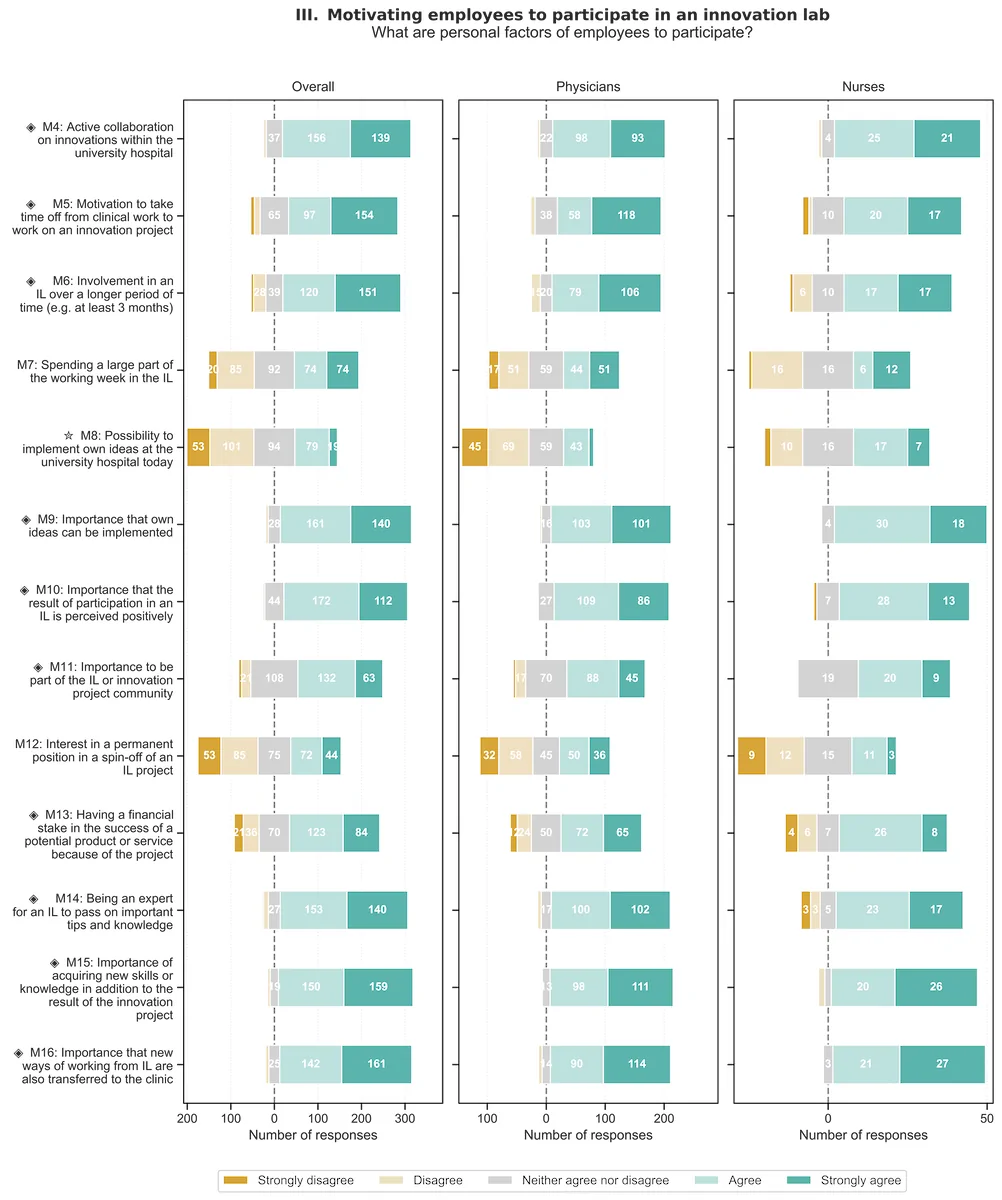
Overall responses to motivating employees’ questions M4 to M16, focusing on personal factors that drive motivation for participating in an innovation lab. Responses marked with diamonds have a median ≥4, and stars indicate statistically significant differences between professional groups. IL: innovation lab.

Respondents identified several motivating factors for participating in innovation initiatives (Figure 6). The highest levels of agreement were reported for acquiring new knowledge and skills (M15: 309/334, 92.51% agreed or strongly agreed), applying new working methods in clinical settings (M16: 303/335, 90.45%), and ensuring that own ideas can be implemented (M9: 301/335, 89.85%). Recognition as an expert and access to collaborative environments were also important, with 87.72% (293/334; M14) agreeing that being available as an expert would motivate participation and 86.76% (295/340; M4) endorsing active collaboration within the hospital.

Additional drivers included the positive perception of IL results (284/333, 85.29%; M10) and the opportunity to take time off from clinical duties (251/338, 74.26%; M5). Lower levels of agreement were found for spending a large part of the working week on IL project commitment (148/345, 42.90%; M7), and only 35.26% (116/329; M12) supported a permanent position in a spin-off company, with 41.95% (138/329) actively disagreeing.

No statistically significant differences were observed between professional groups except for the possibility of implementing one’s own ideas at a university hospital today, which showed a statistically significant difference between physicians and nurses (M8: U=3665.5; *P*<.001), with higher agreement among nurses.

##### Personal Goals for Collaboration (M17-M18)

**Figure 7:**
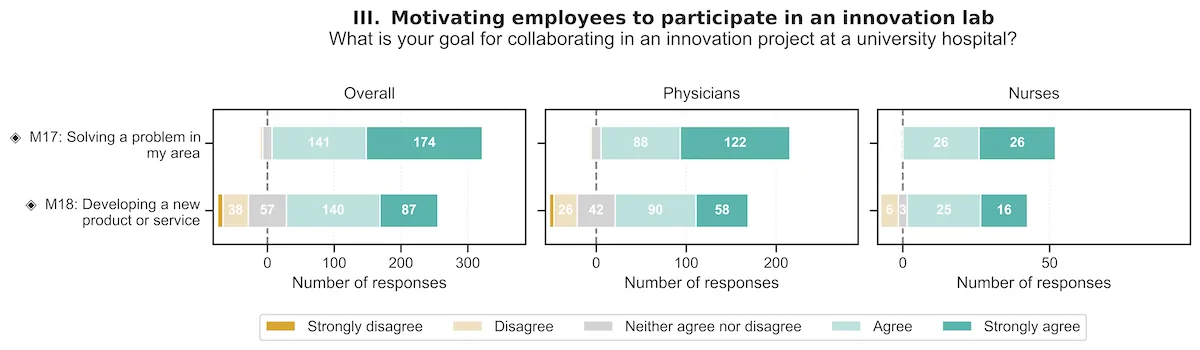
Overall responses to motivating employees’ questions M17 to M18, focusing on personal goals for collaboration in an innovation lab. Responses marked with diamonds have a median ≥4, and stars indicate statistically significant differences between professional groups.

With respect to personal goals for participating in ILs, the most endorsed motivation was solving a clinical problem within one’s own area (M17: 315/333, 94.59% agreed or strongly agreed; Figure 7). This was followed by the goal of developing a new product or service (M18: 227/330, 68.79%). No statistically significant differences between professional groups were observed for either item.

## Discussion

### Principal Findings

This mixed methods study reveals that the successful integration of clinical staff into hospital-based ILs requires strategic alignment with institutional missions, robust structural support, and attention to diverse professional motivations. The findings demonstrate that university hospital staff strongly endorse ILs that are embedded within existing organizational structures and explicitly connected to patient care, research, and education. The preference for internal over external IL models, coupled with the emphasis on workflow improvements over commercial outcomes, suggests that clinical staff view innovation as an extension of their core professional responsibilities rather than as a separate entrepreneurial activity.

The study identified several key enablers of meaningful staff engagement: protected time allocation, adequate funding, access to expertise, and clearly defined processes. Notably, differences between physicians and nurses emerged in several domains, including perceptions of business-oriented innovation, motivation factors, and structural support needs. These findings highlight the importance of tailored engagement strategies that recognize the distinct professional contexts and priorities of different professional groups. A summary of the main quantitative findings is provided in Table 3.

**Table 3:** Summary of key survey findings by typological category. The top 3 findings per category are shown. Group differences were tested using the Mann-Whitney U test. All between-group comparisons are exploratory. Full item-level results are provided in Multimedia Appendix 4.

Category, item, and key finding				Agreement (agreed or strongly agree) or disagreement (disagreed or strongly disagreed), n (%)		Physician vs nurse difference significant?
Category 1: strategic alignment						
	S5	Workflow improvement including digitalization: most endorsed outcome overall	Agreed: 389 (98)		No	
	S1	Patient care as primary mission focus	Agreed: 394 (91.4)		No	
	S4	Business model improvement: lowest endorsement; most opposed item in category	Disagreed: 118 (30.7)		Yes (*P*=.007)	
Category 2: design principles						
	D2	Sufficient funding: top-endorsed condition for participation	Agreed: 337 (95.5)		No	
	D1	Protected working time allocation	Agreed: 336 (95.2)		No	
	D11	External spin-off model: majority opposed	Disagreed: 184 (53.6)		No	
Category 3: motivation						
	M15	Acquiring new knowledge and skills: top motivator	Agreed: 309 (92.5)		No	
	M17	Solving a clinical problem in own area: top personal goal	Agreed: 315 (94.6)		No	
	M12	Permanent spin-off position: lowest endorsement	Agreed: 116 (35.3); disagreed: 138 (42)		No	

### Strategic Alignment Drives Legitimacy and Engagement

The strong preference for ILs that are aligned with the 3 core missions of university hospitals—patient care, research, and education—reflects the institutional identity and values of academic medical centers. This finding aligns with the digital transformation literature in health care organizations, which emphasizes the importance of strategic alignment between innovation initiatives and institutional priorities [2,3]. Unlike private sector innovation labs that may prioritize market opportunities or return on investment, hospital-based ILs must navigate the complex mission of academic medical centers, where clinical excellence, knowledge generation, and education are equally valued.

The lower endorsement of business model improvements and financial outcomes suggests that clinical staff may view purely commercial innovation as potentially misaligned with the fundamental purpose of health care: serving patients. While understandable, this presents a challenge for hospital leaders, who must balance innovation investment with fiscal responsibility. Creating transparent linkages between innovation outcomes and institutional sustainability, including job security, infrastructure improvements, and care quality, may help bridge this gap without compromising professional values.

The strong support for digital transformation and IL projects indicates that staff recognize the need for these advancements in health care delivery [23,24]. This presents an opportunity for hospital leaders to position ILs as integral components of digital transformation strategies rather than peripheral innovation activities.

### Structural Integration as a Foundation for Success

The strong preference for internal IL structures over external spin-off models points to the importance of institutional embedding for health care innovation. External models may face barriers related to trust, contextual relevance, and organizational legitimacy. These challenges map onto Rogers’ Diffusion of Innovations theory [25], particularly the diffusion barriers of complexity and lack of compatibility. Internal labs, embedded within existing institutional structures and cultures, may be better positioned to satisfy these principles, which could account for the higher staff agreement rates.

Successfully embedding an IL also depends on enabling conditions [8]. The emphasis on protected time, adequate funding, and access to expertise underscores the resource-intensive nature of meaningful innovation participation. In hospital settings where clinical care is paramount, innovation activities must be formally supported rather than relegated to discretionary time [26]. Innovation should be considered a strategic priority, not an optional add-on. Without sufficient structural and financial support, IL participation risks being deprioritized in favor of immediate clinical responsibilities.

The requirement for clear processes and legal frameworks reflects the highly regulated nature of health care environments. Unlike technology or consumer product innovation, health care innovation must navigate complex regulatory, ethical, and safety considerations that require explicit organizational support and guidance.

Taken together, structural integration is a practical precondition for the viability of ILs in academic medical centers, rather than merely an organizational preference. Hospitals seeking to establish ILs should therefore prioritize embedding them within existing governance, funding, and legal structures, or creating new ones, from the outset rather than treating structural integration as a downstream implementation detail.

### Professional Diversity Requires Tailored Engagement Strategies

The observed differences between physicians and nurses across multiple survey domains highlight the heterogeneity of health care professionals and their distinct relationships with innovation. Nurses showed greater agreement with business-oriented outcomes and placed different emphasis on conditions for participation and motivation, possibly reflecting their closer connection to operational workflows. Physicians, conversely, may be more focused on research outcomes that align with their training and professional identity.

These differences have practical implications for IL design and staff engagement. Rather than using a one-size-fits-all approach, successful ILs may need to offer multiple participation pathways that accommodate different professional perspectives, time constraints, and motivational factors.

The finding that both groups preferred solving clinical problems within their own areas suggests that successful IL projects should be grounded in real-world clinical challenges rather than technology-driven solutions seeking applications. This aligns with user-centered design principles and emphasizes the importance of clinician involvement from project inception rather than during the implementation phase [27].

### Motivation Patterns Reveal Intrinsic vs Extrinsic Drivers

The strong preference for acquiring new skills, applying novel methods, and solving clinical problems suggests that intrinsic motivational factors are more powerful drivers of innovation participation than extrinsic rewards. This is reinforced by the comparatively low importance placed on financial compensation and a permanent spin-off position, indicating that staff view IL participation as professional development rather than a career transition [28,29].

Consistent with this, the preference for flexible, time-limited involvement over a substantial weekly commitment suggests that clinical staff see innovation as complementary to their primary professional identity rather than a replacement for it. ILs should therefore be designed as platforms for episodic engagement rather than continuous commitment.

The emphasis on seeing the tangible implementation of IL project results may reflect the outcome orientation of health care professionals, who are accustomed to direct patient impact. While staff are motivated by visible outcomes, the uncertainty inherent in innovation processes may create frustration if not properly managed. Clear communication about project timelines, success metrics, and implementation pathways could be an important aspect of sustaining engagement.

### Implications for Hospital Leadership and IL Implementation

These findings have several implications for hospital executives and innovation leaders.

First, ILs should be positioned as strategic investments in organizational capability rather than as experimental initiatives. This positioning requires visible executive support, integration with existing strategic planning processes, and clear communication about how innovation contributes to institutional mission fulfillment.

Second, the resource requirements for meaningful staff engagement, particularly protected time and adequate funding, necessitate formal budget allocation and staffing model adjustments. Organizations should view these investments as essential infrastructure for innovation capability rather than optional enhancements. The long-term return on investment may include improved care delivery, enhanced staff satisfaction, and increased organizational adaptability.

Third, the diversity of professional perspectives and motivational factors requires engagement strategies that go beyond generic innovation programs. Successful ILs will likely need to offer multiple participation models, project types, and recognition mechanisms that align with different professional cultures within the hospital.

### Limitations

Several limitations should be considered when interpreting these findings. The single-center design restricts generalizability, as the findings reflect the organizational context of 1 large German university hospital. Charité’s size, academic mandate, innovation culture, and resource base may not be representative of those of smaller or nonacademic hospitals, and the German health care context, with its specific funding mechanisms, professional hierarchies, and labor structures, may limit transferability to other national systems. International comparative studies would help distinguish universal from context-specific determinants of IL engagement. Furthermore, Charité’s preexisting innovation infrastructure may have shaped staff perceptions and inflated endorsement of IL-oriented survey items relative to institutions at earlier stages of innovation maturity. After data collection was completed, the Charité BIH ARC Innovation Center was established as a new central reference point for innovation activities at Charité, including an IL structure, which lends further credibility to the local applicability of our findings.

Sampling bias is a further concern. Recruitment through institutional mailing lists and voluntary participation likely overrepresented staff with prior interest in or exposure to innovation, potentially inflating perceived receptiveness to ILs. The uneven distribution of professional groups represents a notable limitation: physicians constituted 67.7% (226/334) of respondents, while nurses accounted for only 15.9% (53/334), despite nurses comprising more than 50% of Charité’s clinical workforce (approximately 6900 nurses vs 5700 physicians and researchers). This imbalance reflects both structural recruitment differences and cultural factors. Most proximately, whereas physicians were reached via a Charité-wide mailing list enabling direct contact, no equivalent list existed for nursing staff; the survey instead had to be forwarded by nursing team leads. This indirect recruitment chain likely suppressed nurse response rates independently of any cultural factors. Additionally, physicians’ greater familiarity with academic research participation, stronger perceived relevance of institutional research to their professional role, and more frequent direct use of institutional email channels may have further contributed to the imbalance. Aggregate findings may consequently be skewed toward medical perspectives, and between-group comparisons involving nurses should be interpreted with caution. Additionally, department-level data (eg, surgical vs nonsurgical affiliation) were not collected, precluding subgroup analyses at that level.

The cross-sectional survey design prevents causal inference about the relationship between IL characteristics and staff engagement. Combined with the reliance on self-reported perceptions rather than observed behavior or actual participation data, the findings reflect stated attitudes rather than demonstrated engagement. How these preferences translate into action under real organizational conditions remains an open question.

### Future Work

Several research opportunities emerge from this study. Longitudinal research could examine how staff perceptions and engagement evolve as ILs mature and produce tangible outcomes. Interventional studies comparing different IL models, participation formats, or support structures could provide evidence about effective implementation strategies.

Comparative studies across different types of health care organizations, such as community hospitals, health systems, and university hospitals, could identify how organizational context influences IL success factors. Similarly, international comparisons could illuminate how health care system characteristics and professional cultures affect innovation participation.

Future studies should use targeted recruitment strategies to ensure more balanced representation of professional groups and further examine the perspectives of nonclinical staff, including administrators, technicians, and support personnel, whose roles are integral to health care innovation but were underrepresented in this study. Understanding how IL participation affects professional identity, job satisfaction, and organizational commitment could provide insights into long-term sustainability. Differences among professional groups in culture, workflows, and innovation engagement warrant further investigation; therefore, department-level data or surgical and nonsurgical affiliation should be included in future iterations of this survey.

Finally, research examining the actual outcomes of IL participation, including innovation success rates, implementation effectiveness, and organizational impact, would help validate the theoretical frameworks and practical recommendations emerging from this work.

### Conclusions

Clinical staff express willingness to engage meaningfully in university hospital-based innovation labs when 3 conditions are present: strategic alignment with hospital missions, structural integration within hospital operations, and tailored engagement strategies in recognition of diverse professional motivations. Staff perspectives indicate a strong preference for internal models over external or spin-off approaches and for initiatives grounded in workflow improvement and clinical problems within their own areas of practice. Based on these findings, hospital innovation labs may be more effective when positioned as internal problem-solving platforms rather than external incubators. While organizations considering this model should anticipate substantial structural, financial, and stakeholder commitments, this investment may yield substantial returns through enhanced care delivery, improved staff satisfaction, and strengthened organizational adaptability in an increasingly complex health care environment.

## Data Availability

The datasets generated and analyzed during this study are not publicly available due to the conditions of the ethical approval but are available from the corresponding author on reasonable request.

## Appendix

Multimedia Appendix 1 Reporting guidelines.

Multimedia Appendix 2 Interview guide for expert interviews with external experts and hospital management.

Multimedia Appendix 3 English translation of the survey.

Multimedia Appendix 4 Survey findings.

Multimedia Appendix 5 Overall responses to questions S1 to S3.

Multimedia Appendix 6 Overall responses to questions S7 to S8.

Multimedia Appendix 7 Overall responses to questions D1 to D9.

Multimedia Appendix 8 Overall responses to questions M1 to M3.

## Abbreviations

CROSS: Checklist for Reporting of Survey Studies
IL: innovation lab
SRQR: Standards for Reporting Qualitative Research
