# Supplementary material for: Clinician Participation in Innovation Labs at University Hospitals: Mixed Methods Study
Source: J Med Internet Res. 2026 Aug 7;28:e84887. doi: 10.2196/84887 (PMC13498907; doi:10.2196/84887)
Supplement: Supplementary file 2 [file jmir_v28i1e84887_app2.docx]

**Multimedia Appendix 2:** Interview guide for expert interviews with external experts and hospital management.

| **ID** | **Addressee** | **Question** |
| --- | --- | --- |
| E1 | External experts | What is your innovation lab’s goal for innovation (products, processes, digitalization, etc.) within the value chain of your industry sector and why? |
| E2 | External experts | How is your innovation lab interlinked with the organization, and how are stakeholders (human resources, communications department, etc.) involved? Are there different types of collaboration with the specialist departments in your Innovation lab? |
| E3 | External experts | How does collaboration take place between your innovation lab and internal employees and stakeholders from the specialist departments? Are employees in regular contact with their innovation lab? |
| E4 | External experts | How does the collaboration between your Innovation la and an external partner work? |
| E5 | External experts | Which innovation methods and innovation processes (e.g., idea platforms, hackathons, etc.) are used in your innovation lab, and which are particularly successful? What are the values practiced in your innovation lab? |
| E6 | External experts | How is your innovation lab staffed? What roles and competencies are there in the team? |
| E7 | External experts | What processes, technologies, and decision-making channels are established within the team? |
| E8 | External experts | How do you create the freedom and framework conditions for internal employees to develop new innovations within the innovation lab? Which framework conditions are fundamental? |
| E9 | External experts | How do you achieve a sustainably high level of motivation and commitment to factors of motivation in organizations, such as Positive Outcome, Community / Organization, Human / Employee, and Human needs of employees from the specialist departments, for participation in the innovation lab? |
| E10 | External experts | What incentives do you see for employees to participate in the innovation lab? |
| E11 | External experts | What are the main challenges (organizational, legal, technological) for the establishment and sustainable operation of an innovation lab from your point of view? How did you overcome them? What do you see as particularly relevant for cooperation with the specialist departments? |
| H1 | Hospital management | What opportunities and challenges do you see for an innovation lab at Charité here? What inspires you personally, and what reservations might you have about the local innovation lab? |
| H2 | Hospital management | Which areas of innovation should be prioritized in teaching, patient care, or research? When do you consider these to have been successfully addressed? |
| H3 | Hospital management | Is there a prioritization for your clinical employees from your department to work on innovations in a local innovation lab? For example, how do you view collaboration on innovation that does not (directly) contribute to your departmental goals? |
| H4 | Hospital management | What framework conditions would be conducive to cooperation between clinical staff and a local innovation lab? |
| H5 | Hospital management | How do you think collaboration with a local innovation lab at Charité can be integrated into everyday clinical practice? |
| H6 | Hospital management | What expectations do you have of the transparency and communication of the local innovation lab? |
| H7 | Hospital management | How can a local innovation lab motivate clinical staff to collaborate and achieve high engagement in organizational motivation factors such as positive outcome, community/organization, human/employee, and human needs? For example: How deeply should they be involved in the development of innovations? |
| H8 | Hospital management | Which innovation methods (e.g., idea platforms, hackathons, etc.) should a local innovation lab offer? Which approaches from other clinics do you know, and do you think are interesting? |
| H9 | Hospital management | Which groups of clinical employees do you think are particularly relevant and motivated to work in a local innovation lab? Conversely, which would be particularly relevant and need to be motivated? |
| H10 | Hospital management | In your opinion, what framework conditions (organizational, legal, technological) must a local innovation lab offer for cooperation with your department? |
| Note: Original items were in German and have been translated to English. | | |
