# Supplementary material for: Clinician Participation in Innovation Labs at University Hospitals: Mixed Methods Study
Source: J Med Internet Res. 2026 Aug 7;28:e84887. doi: 10.2196/84887 (PMC13498907; doi:10.2196/84887)
Supplement: Supplementary file 3 [file jmir_v28i1e84887_app3.docx]

**Multimedia Appendix 3**: English translation of the survey.

| Typological category | Question | ID | Item | Response options |
| --- | --- | --- | --- | --- |
| I. Strategic alignment of innovation labs | Which of the three main tasks of the university hospital should form the focus of an innovation lab in your view? | S1 | Patient care | 5-Point-Likert options^a^ |
|  |  | S2 | Education | 5-Point-Likert options^a^ |
|  |  | S3 | Research | 5-Point-Likert options^a^ |
|  | What kind of ideas should be developed in an innovation lab at a university hospital? | S4 | Tools or measures for improving business outcomes (profit increase and cost reduction) | 5-Point-Likert options^a^ |
|  |  | S5 | Improvements to workflows (including digitalization of processes) | 5-Point-Likert options^a^ |
|  |  | S6 | New products and services for clinical problems (including AI, apps and sensors, or blockchain) | 5-Point-Likert options^a^ |
|  | What should a university hospital's innovation lab focus on? | S7 | Internal innovation (e.g. in-house development within the university hospital) | 5-Point-Likert options^a^ |
|  |  | S8 | External collaboration (e.g. collaboration with external startups) | 5-Point-Likert options^a^ |
|  | What aspects should the innovation lab strategically support? | S9 | Strengthening the digital transformation of the university hospital | 5-Point-Likert options^a^ |
|  |  | S10 | Promoting employees with entrepreneurial aspirations within the university hospital | 5-Point-Likert options^a^ |
|  |  | S11 | Integrating external products or services from the market into the university hospital | 5-Point-Likert options^a^ |
|  |  | S12 | Providing a platform or network for the exchange of innovation topics | 5-Point-Likert options^a^ |
|  |  | S13 | Creating awareness through regular hospital-wide communication within the university hospital | 5-Point-Likert options^a^ |
|  |  | S14 | Carrying out innovation projects within the university hospital | 5-Point-Likert options^a^ |
|  |  | S15 | Adapting corporate culture within the university hospital | 5-Point-Likert options^a^ |
| II. Design principles of innovation labs | In your opinion, which of the following conditions are important for participation in the innovation lab? | D1 | Protected time for participation | 5-Point-Likert options^a^ |
|  |  | D2 | Sufficient funding | 5-Point-Likert options^a^ |
|  |  | D3 | Dedicated place for collaboration | 5-Point-Likert options^a^ |
|  |  | D4 | Access to necessary skills or experts (external if necessary) | 5-Point-Likert options^a^ |
|  |  | D5 | Organization and processes of the innovation project | 5-Point-Likert options^a^ |
|  |  | D6 | Legal aspects and requirements | 5-Point-Likert options^a^ |
|  |  | D7 | Linking with the various business areas of the university hospital | 5-Point-Likert options^a^ |
|  |  | D8 | Modern forms of collaboration (e.g. agile working or design thinking) | 5-Point-Likert options^a^ |
|  |  | D9 | Financial compensation | 5-Point-Likert options^a^ |
|  | How should the innovation lab be organized within the university hospital? | D10 | As a department of the university hospital | 5-Point-Likert options^a^ |
|  |  | D11 | As an external company (spin-off from the university hospital) | 5-Point-Likert options^a^ |
| III. Motivating employees to participate in an innovation lab | Which of the university hospital’s three main tasks would motivate you to participate in an innovation project? | M1 | Patient care | 5-Point-Likert options^a^ |
|  |  | M2 | Education | 5-Point-Likert options^a^ |
|  |  | M3 | Research | 5-Point-Likert options^a^ |
|  | What are personal factors of employees to participate? | M4 | Active collaboration on innovations within the university hospital | 5-Point-Likert options^a^ |
|  |  | M5 | Motivation to take time off from clinical work to work on an innovation project | 5-Point-Likert options^a^ |
|  |  | M6 | Involvement in an IL over a longer period of time (e.g. at least 3 months) | 5-Point-Likert options^a^ |
|  |  | M7 | Spending a large part of the working week in the IL | 5-Point-Likert options^a^ |
|  |  | M8 | Possibility to implement own ideas at the university hospital today | 5-Point-Likert options^a^ |
|  |  | M9 | Importance that own ideas can be implemented | 5-Point-Likert options^a^ |
|  |  | M10 | Importance that the result of participation in an IL is perceived positively | 5-Point-Likert options^a^ |
|  |  | M11 | Importance to be part of the IL or innovation project community | 5-Point-Likert options^a^ |
|  |  | M12 | Interest in a permanent position in a spin-off of an IL project | 5-Point-Likert options^a^ |
|  |  | M13 | Having a financial stake in the success of a potential product or service because of the project | 5-Point-Likert options^a^ |
|  |  | M14 | Being an expert for an IL to pass on important tips and knowledge | 5-Point-Likert options^a^ |
|  |  | M15 | Importance of acquiring new skills or knowledge in addition to the result of the innovation project | 5-Point-Likert options^a^ |
|  |  | M16 | Importance that new ways of working from IL are transferred to the clinic | 5-Point-Likert options^a^ |
|  | What is your goal for collaborating in an innovation project at a university hospital? | M17 | Solving a problem in my area | 5-Point-Likert options^a^ |
|  |  | M18 | Developing a new product or service | 5-Point-Likert options^a^ |
| Demographic questions | | G1 | Gender | Female, male, nonbinary, prefer not to say |
|  |  | G2 | Years of professional experience | 0-5 years, 6-10 years, >10 years |
|  |  | G3 | Primary responsibilities | Patient care, research, education |
|  |  | G4 | Profession | Nurse, physician, other health care professional, trainee/student (medical/nursing), other |
| ^a^ Response options and the corresponding coding for data analysis (in brackets) is: "Strongly disagree" (1), "Disagree" (2), "Neither agree nor disagree" (3), "Agree" (4), "Strongly agree" (5). IL: innovation lab.  Note: Original items were in German and have been translated to English. Local context (“Charité”) has been reworded (“university hospital”). | | | | |
