# Supplementary material for: Clinician Participation in Innovation Labs at University Hospitals: Mixed Methods Study
Source: J Med Internet Res. 2026 Aug 7;28:e84887. doi: 10.2196/84887 (PMC13498907; doi:10.2196/84887)
Supplement: Supplementary file 4 [file jmir_v28i1e84887_app4.docx]

**Multimedia Appendix 4:** Survey findings.

Survey items S1-S3.

| Res^a^ | Pop | N | x͂ | SD | IQR | 95% CI | Var | Kurt | Skew |
| --- | --- | --- | --- | --- | --- | --- | --- | --- | --- |
| ◈ S1 | O | 431 | 5 | 0.78 | 1 (4 to 5) | 4.49 (4.42 to 4.57) | 0.61 | 3.67 | -1.83 |
| S1 | P | 224 | 5 | 0.79 | 1 (4 to 5) | 4.49 (4.39 to 4.6) | 0.63 | 4.14 | -1.92 |
| S1 | N | 51 | 5 | 0.5 | 1 (4 to 5) | 4.69 (4.54 to 4.83) | 0.26 | 0.49 | -1.25 |
| ◈ S2 | O | 409 | 4 | 0.89 | 1 (4 to 5) | 4.02 (3.94 to 4.11) | 0.79 | 1.09 | -1.01 |
| S2 | P | 214 | 4 | 0.91 | 1 (4 to 5) | 3.93 (3.81 to 4.05) | 0.83 | 0.58 | -0.84 |
| S2 | N | 45 | 4 | 0.89 | 1 (4 to 5) | 4,09 (3.82 to 4.36) | 0.81 | 3.99 | -1.69 |
| ◈ S3 | O | 413 | 4 | 0.79 | 1 (4 to 5) | 4.3 (4.23 to 4.38) | 0.63 | 1.88 | -1.24 |
| S3 | P | 217 | 5 | 0.79 | 1 (4 to 5) | 4.35 (4.25 to 4.46) | 0.63 | 0.9 | -1.17 |
| S3 | N | 45 | 4 | 0.98 | 1 (4 to 5) | 4.09 (3.79 to 4.39) | 0.99 | 3.76 | -1.85 |
| Res = Responses; Pop = Population (O = overall, P = physicians, N = nurses); N = Number of observations; x͂ = Median; SD = Standard Deviation; IQR = Interquartile Range; 95% CI = 95% Confidence Interval; Var = Variance; Kurt = Kurtosis; Skew = Skewness.  ^a^ Responses marked with diamonds (◈) have a median ≥ 4; Responses marked with stars (✮) indicate statistically significant differences between professional groups. | | | | | | | | | |

Survey items S4-S6.

| Res^a^ | Pop^b^ | N | x͂ | SD | IQR | 95% CI | Var | Kurt | Skew |
| --- | --- | --- | --- | --- | --- | --- | --- | --- | --- |
| S4 | O | 385 | 3 | 1.15 | 2 (2 to 4) | 3.09 (2.98 to 3.21) | 1.32 | -0.82 | -0.31 |
| ✮ S4 | P | 220 | 3 | 1.13 | 2 (2 to 4) | 2.99 (2.84 to 3.14) | 1.28 | -0.81 | -0.21 |
| ✮ S4 | N | 47 | 4 | 1.07 | 1 (3 to 4) | 3.57 (3.26 to 3.89) | 1.16 | 0.17 | -0.88 |
| ◈ S5 | O | 397 | 5 | 0.51 | 1 (4 to 5) | 4.69 (4.64 to 4.74) | 0.26 | 1.91 | -1.48 |
| S5 | P | 225 | 5 | 0.53 | 1 (4 to 5) | 4.71 (4.64 to 4.78) | 0.28 | 3.47 | -1.82 |
| S5 | N | 52 | 5 | 0.42 | 0 (5 to 5) | 4.77 (4.65 to 4.89) | 0.18 | -0.37 | -1.28 |
| ◈ S6 | O | 394 | 4 | 0.82 | 1 (4 to 5) | 4.28 (4.2 to 4.36) | 0.67 | 1.81 | -1.23 |
| S6 | P | 225 | 4 | 0.87 | 1 (4 to 5) | 4.28 (4.16 to 4.39) | 0.76 | 2.3 | -1.41 |
| S6 | N | 50 | 4.5 | 0.66 | 1 (4 to 5) | 4.4 (4.21 to 4.59) | 0.45 | -0.63 | -0.66 |
| Res = Responses; Pop = Population (O = overall, P = physicians, N = nurses); N = Number of observations; x͂ = Median; SD = Standard Deviation; IQR = Interquartile Range; 95% CI = 95% Confidence Interval; Var = Variance; Kurt = Kurtosis; Skew = Skewness. ^a^ Responses marked with diamonds (◈) have a median ≥ 4; Responses marked with stars (✮) indicate statistically significant differences between professional groups. | | | | | | | | | |

Survey items S7-S8.

| Resp^a^ | Pop^b^ | N | x͂ | SD | IQR | 95% CI | Var | Kurt | Skew |
| --- | --- | --- | --- | --- | --- | --- | --- | --- | --- |
| ◈ S7 | O | 386 | 5 | 0.78 | 1 (4 to 5) | 4.39 (4.32 to 4.47) | 0.61 | 4.24 | -1.76 |
| S7 | P | 222 | 5 | 0.86 | 1 (4 to 5) | 4.35 (4.23 to 4.46) | 0.73 | 2.97 | -1.64 |
| S7 | N | 52 | 5 | 0.69 | 1 (4 to 5) | 4.46 (4.27 to 4.66) | 0.49 | 9.3 | -2.3 |
| ◈ S8 | O | 381 | 4 | 0.93 | 2 (3 to 5) | 3.91 (3.82 to 4.01) | 0.86 | 0.6 | -0.84 |
| S8 | P | 224 | 4 | 0.88 | 1 (4 to 5) | 3.99 (3.87 to 4.1) | 0.78 | 0.4 | -0.76 |
| S8 | N | 46 | 4 | 0.88 | 1 (4 to 5) | 4.04 (3.78 to 4.31) | 0.8 | 0.15 | -0.84 |
| Res = Responses; Pop = Population (O = overall, P = physicians, N = nurses); N = Number of observations; x͂ = Median; SD = Standard Deviation; IQR = Interquartile Range; 95% CI = 95% Confidence Interval; Var = Variance; Kurt = Kurtosis; Skew = Skewness. ^a^ Responses marked with diamonds (◈) have a median ≥ 4; Responses marked with stars (✮) indicate statistically significant differences between professional groups. | | | | | | | | | |

Survey items S9-S15.

| **Resp^a^** | **Pop^b^** | **N** | **x͂** | **SD** | **IQR** | **95% CI** | **Var** | **Kurt** | **Skew** |
| --- | --- | --- | --- | --- | --- | --- | --- | --- | --- |
| ◈ S9 | O | 365 | 5 | 0.59 | 1 (4 to 5) | 4.53 (4.46 to 4.59) | 0.35 | 0.8 | -1 |
| S9 | P | 224 | 5 | 0.57 | 1 (4 to 5) | 4.59 (4.52 to 4.67) | 0.32 | 1.2 | -1.18 |
| S9 | N | 51 | 5 | 0.54 | 1 (4 to 5) | 4.55 (4.4 to 4.7) | 0.29 | -0.89 | -0.58 |
| ◈ S10 | O | 364 | 4 | 1.12 | 1 (3 to 4) | 3.58 (3.46 to 3.69) | 1.25 | -0.54 | -0.43 |
| ✮ S10 | P | 222 | 4 | 1.14 | 1 (3 to 4) | 3.54 (3.39 to 3.69) | 1.31 | -0.73 | -0.35 |
| ✮ S10 | N | 52 | 4 | 0.98 | 1.25 (3.75 to 5) | 4.04 (3.76 to 4.31) | 0.98 | 0.43 | -0.94 |
| ◈ S11 | O | 365 | 4 | 0.9 | 1 (3 to 4) | 3.62 (3.53 to 3.71) | 0.81 | 0.65 | -0.71 |
| S11 | P | 224 | 4 | 0.92 | 1 (3 to 4) | 3.63 (3.51 to 3.76) | 0.85 | 0.75 | -0.79 |
| S11 | N | 49 | 4 | 0.82 | 1 (3 to 4) | 3.82 (3.58 to 4.06) | 0.69 | -0.11 | -0.52 |
| ◈ S12 | O | 368 | 4 | 0.78 | 1 (4 to 5) | 4.12 (4.04 to 4.2) | 0.61 | 1.08 | -0.86 |
| S12 | P | 226 | 4 | 0.83 | 1 (4 to 5) | 4.08 (3.98 to 4.19) | 0.69 | 1.15 | -0.95 |
| S12 | N | 51 | 4 | 0.77 | 1 (4 to 5) | 4.2 (3.98 to 4.41) | 0.6 | -0.29 | -0.61 |
| ◈ S13 | O | 367 | 4 | 0.78 | 1 (4 to 5) | 4.17 (4.09 to 4.25) | 0.61 | 0.98 | -0.86 |
| ✮ S13 | P | 225 | 4 | 0.84 | 1 (4 to 5) | 4.08 (3.97 to 4.19) | 0.7 | 0.87 | -0.87 |
| ✮ S13 | N | 51 | 4 | 0.57 | 1 (4 to 5) | 4.45 (4.29 to 4.61) | 0.33 | -0.76 | -0.44 |
| ◈ S14 | O | 367 | 5 | 0.61 | 1 (4 to 5) | 4.44 (4.38 to 4.51) | 0.37 | 0.33 | -0.76 |
| S14 | P | 226 | 5 | 0.59 | 1 (4 to 5) | 4.48 (4.4 to 4.56) | 0.35 | 0.32 | -0.78 |
| S14 | N | 51 | 4 | 0.72 | 1 (4 to 5) | 4.27 (4.07 to 4.48) | 0.52 | 0.42 | -0.78 |
| ◈ S15 | O | 368 | 4 | 0.86 | 1 (4 to 5) | 4.04 (3.95 to 4.13) | 0.74 | 0.57 | -0.77 |
| S15 | P | 226 | 4 | 0.89 | 2 (3 to 5) | 3.97 (3.85 to 4.09) | 0.79 | 0.37 | -0.69 |
| S15 | N | 50 | 4 | 0.62 | 1 (4 to 5) | 4.24 (4.06 to 4.42) | 0.39 | -0.59 | -0.21 |
| Res = Responses; Pop = Population (O = overall, P = physicians, N = nurses); N = Number of observations; x͂ = Median; SD = Standard Deviation; IQR = Interquartile Range; 95% CI = 95% Confidence Interval; Var = Variance; Kurt = Kurtosis; Skew = Skewness. ^a^ Responses marked with diamonds (◈) have a median ≥ 4; Responses marked with stars (✮) indicate statistically significant differences between professional groups. | | | | | | | | | |

Survey items D1-D9.

| Res^a^ | Pop^b^ | N | x͂ | SD | IQR | 95% CI | Var | Kurt | Skew |
| --- | --- | --- | --- | --- | --- | --- | --- | --- | --- |
| ◈ D1 | O | 353 | 5 | 0.61 | 1 (4 to 5) | 4.56 (4.5 to 4.63) | 0.38 | 1.63 | -1.3 |
| D1 | P | 223 | 5 | 0.63 | 1 (4 to 5) | 4.57 (4.49 to 4.66) | 0.4 | 1.76 | -1.41 |
| D1 | N | 51 | 5 | 0.63 | 1 (4 to 5) | 4.57 (4.39 to 4.75) | 0.41 | 3.36 | -1.64 |
| ◈ D2 | O | 353 | 5 | 0.61 | 1 (4 to 5) | 4.49 (4.43 to 4.56) | 0.38 | 1.07 | -1.01 |
| D2 | P | 223 | 5 | 0.59 | 1 (4 to 5) | 4.54 (4.46 to 4.62) | 0.35 | 0.73 | -1.02 |
| D2 | N | 52 | 4 | 0.72 | 1 (4 to 5) | 4.31 (4.1 to 4.51) | 0.53 | 1.81 | -1.15 |
| ◈ D3 | O | 350 | 5 | 0.66 | 1 (4 to 5) | 4.43 (4.36 to 4.5) | 0.43 | 1.49 | -1.09 |
| D3 | P | 219 | 5 | 0.71 | 1 (4 to 5) | 4.41 (4.32 to 4.51) | 0.51 | 1.66 | -1.24 |
| D3 | N | 52 | 5 | 0.57 | 1 (4 to 5) | 4.5 (4.34 to 4.66) | 0.33 | -0.62 | -0.62 |
| ◈ D4 | O | 354 | 4 | 0.68 | 1 (4 to 5) | 4.36 (4.29 to 4.43) | 0.46 | 0.67 | -0.86 |
| D4 | P | 224 | 4 | 0.71 | 1 (4 to 5) | 4.36 (4.26 to 4.45) | 0.5 | 0.68 | -0.94 |
| D4 | N | 51 | 4 | 0.59 | 1 (4 to 5) | 4.37 (4.2 to 4.54) | 0.36 | -0.69 | -0.35 |
| ◈ D5 | O | 347 | 4 | 0.77 | 1 (4 to 5) | 4.2 (4.12 to 4.28) | 0.6 | 1.03 | -0.89 |
| D5 | P | 222 | 4 | 0.78 | 1 (4 to 5) | 4.2 (4.1 to 4.31) | 0.61 | 0.83 | -0.88 |
| D5 | N | 50 | 4 | 0.63 | 1 (4 to 5) | 4.4 (4.22 to 4.58) | 0.41 | -0.62 | -0.57 |
| ◈ D6 | O | 351 | 4 | 0.81 | 1 (4 to 5) | 4.11 (4.02 to 4.19) | 0.66 | 0.58 | -0.77 |
| D6 | P | 224 | 4 | 0.87 | 1 (4 to 5) | 4.08 (3.97 to 4.2) | 0.77 | 0.51 | -0.85 |
| D6 | N | 51 | 4 | 0.6 | 1 (4 to 5) | 4.27 (4.11 to 4.44) | 0.36 | -0.57 | -0.19 |
| ◈ D7 | O | 352 | 4 | 0.87 | 1 (4 to 5) | 4 (3.91 to 4.09) | 0.76 | 0.4 | -0.75 |
| ✮ D7 | P | 223 | 4 | 0.85 | 1.5 (3 to 4.5) | 3.92 (3.81 to 4.03) | 0.73 | 0.41 | -0.67 |
| ✮ D7 | N | 51 | 5 | 0.5 | 1 (4 to 5) | 4.55 (4.41 to 4.69) | 0.25 | -1.96 | -0.2 |
| ◈ D8 | O | 354 | 4 | 0.96 | 2 (3 to 5) | 4 (3.9 to 4.1) | 0.92 | 0.47 | -0.86 |
| ✮ D8 | P | 225 | 4 | 0.97 | 2 (3 to 5) | 3.92 (3.79 to 4.04) | 0.94 | 0.13 | -0.74 |
| ✮ D8 | N | 51 | 5 | 0.72 | 1 (4 to 5) | 4.41 (4.21 to 4.62) | 0.53 | -0.68 | -0.8 |
| ◈ D9 | O | 352 | 4 | 0.91 | 2 (3 to 5) | 3.91 (3.81 to 4) | 0.83 | 0.31 | -0.64 |
| D9 | P | 224 | 4 | 0.91 | 2 (3 to 5) | 3.86 (3.74 to 3.98) | 0.84 | 0.23 | -0.59 |
| D9 | N | 51 | 4 | 0.72 | 1 (4 to 5) | 4.22 (4.01 to 4.42) | 0.53 | -1.04 | -0.35 |
| Res = Responses; Pop = Population (O = overall, P = physicians, N = nurses); N = Number of observations; x͂ = Median; SD = Standard Deviation; IQR = Interquartile Range; 95% CI = 95% Confidence Interval; Var = Variance; Kurt = Kurtosis; Skew = Skewness. ^a^ Responses marked with diamonds (◈) have a median ≥ 4; Responses marked with stars (✮) indicate statistically significant differences between professional groups. | | | | | | | | | |

Survey items D10-D11.

| Res^a^ | Pop^b^ | N | x͂ | SD | IQR | 95% CI | Var | Kurt | Skew |
| --- | --- | --- | --- | --- | --- | --- | --- | --- | --- |
| ◈ D10 | O | 348 | 4 | 1.03 | 2 (3 to 5) | 3.93 (3.82 to 4.04) | 1.06 | 0.19 | -0.85 |
| D10 | P | 224 | 4 | 1.01 | 2 (3 to 5) | 3.93 (3.8 to 4.07) | 1.03 | 0.27 | -0.84 |
| D10 | N | 52 | 4 | 0.95 | 2 (3 to 5) | 3.98 (3.71 to 4.25) | 0.92 | -0.53 | -0.63 |
| D11 | O | 343 | 2 | 1.26 | 1 (2 to 3) | 2.56 (2.43 to 2.69) | 1.6 | -0.86 | 0.43 |
| D11 | P | 223 | 2 | 1.27 | 1.5 (1.5 to 3) | 2.52 (2.36 to 2.69) | 1.61 | -0.78 | 0.49 |
| D11 | N | 48 | 3 | 1.27 | 2 (2 to 4) | 2.73 (2.36 to 3.1) | 1.65 | -1.26 | 0.03 |
| Res = Responses; Pop = Population (O = overall, P = physicians, N = nurses); N = Number of observations; x͂ = Median; SD = Standard Deviation; IQR = Interquartile Range; 95% CI = 95% Confidence Interval; Var = Variance; Kurt = Kurtosis; Skew = Skewness. ^a^ Responses marked with diamonds (◈) have a median ≥ 4; Responses marked with stars (✮) p ≤ .05 indicate statistically significant differences between professional groups. | | | | | | | | | |

Survey items M1-M3.

| Res^a^ | Pop^b^ | N | x͂ | SD | IQR | 95% CI | Var | Kurt | Skew |
| --- | --- | --- | --- | --- | --- | --- | --- | --- | --- |
| ◈ M1 | O | 344 | 5 | 0.86 | 1 (4 to 5) | 4.43 (4.34 to 4.52) | 0.74 | 3.23 | -1.77 |
| M1 | P | 222 | 5 | 0.8 | 1 (4 to 5) | 4.48 (4.38 to 4.59) | 0.64 | 4.42 | -1.93 |
| M1 | N | 53 | 5 | 0.44 | 1 (4 to 5) | 4.74 (4.61 to 4.86) | 0.2 | -0.86 | -1.07 |
| ◈ M2 | O | 336 | 4 | 1.1 | 1 (3 to 4) | 3.54 (3.42 to 3.66) | 1.21 | -0.3 | -0.6 |
| M2 | P | 217 | 4 | 1.08 | 1 (3 to 4) | 3.52 (3.37 to 3.66) | 1.17 | -0.25 | -0.63 |
| M2 | N | 49 | 3 | 1.09 | 1 (3 to 4) | 3.31 (2.99 to 3.62) | 1.22 | -0.15 | -0.63 |
| ◈ M3 | O | 345 | 4 | 1.11 | 2 (3 to 5) | 3.86 (3.74 to 3.97) | 1.23 | -0.3 | -0.74 |
| ✮ M3 | P | 222 | 4 | 1.08 | 2 (3 to 5) | 3.98 (3.83 to 4.12) | 1.17 | 0.2 | -0.96 |
| ✮ M3 | N | 50 | 3 | 1.05 | 1 (3 to 4) | 3.32 (3.02 to 3.62) | 1.12 | -0.53 | -0.04 |
| Res = Responses; Pop = Population (O = overall, P = physicians, N = nurses); N = Number of observations; x͂ = Median; SD = Standard Deviation; IQR = Interquartile Range; 95% CI = 95% Confidence Interval; Var = Variance; Kurt = Kurtosis; Skew = Skewness. ^a^ Responses marked with diamonds (◈) have a median ≥ 4; Responses marked with stars (✮) indicate statistically significant differences between professional groups. | | | | | | | | | |

Survey items M4-M16.

| Res^a^ | Pop^b^ | N | x͂ | SD | IQR | 95% CI | Var | Kurt | Skew |
| --- | --- | --- | --- | --- | --- | --- | --- | --- | --- |
| ◈ M4 | O | 340 | 4 | 0.76 | 1 (4 to 5) | 4.25 (4.17 to 4.33) | 0,58 | 1.46 | -1.01 |
| M4 | P | 219 | 4 | 0.78 | 1 (4 to 5) | 4.26 (4.16 to 4.36) | 0.62 | 2.04 | -1.18 |
| M4 | N | 51 | 4 | 0.69 | 1 (4 to 5) | 4.29 (4.1 to 4.49) | 0.49 | 0.74 | -0.82 |
| ◈ M5 | O | 338 | 4 | 1.01 | 2 (3 to 5) | 4.11 (4 to 4.22) | 1.03 | 0.6 | -1.05 |
| M5 | P | 222 | 5 | 0.89 | 1 (4 to 5) | 4.28 (4.17 to 4.4) | 0.79 | 0.19 | -1.01 |
| M5 | N | 50 | 4 | 0.99 | 1.75 (3.25 to 5) | 3.98 (3.7 to 4.26) | 1 | 1.18 | -1.07 |
| ◈ M6 | O | 344 | 4 | 1.01 | 1 (4 to 5) | 4.11 (4 to 4.22) | 1.03 | 0.53 | -1.1 |
| M6 | P | 222 | 4 | 0.93 | 1 (4 to 5) | 4.23 (4.1 to 4.35) | 0.87 | 0.99 | -1.23 |
| M6 | N | 51 | 4 | 1.07 | 2 (3 to 5) | 3.84 (3.54 to 4.15) | 1.17 | -0.49 | -0.64 |
| M7 | O | 345 | 3 | 1.21 | 2 (2 to 4) | 3.28 (3.15 to 3.41) | 1.48 | -1.08 | -0.02 |
| M7 | P | 222 | 3 | 1.26 | 2 (2 to 4) | 3.27 (3.11 to 3.44) | 1.58 | -1.09 | -0.06 |
| M7 | N | 51 | 3 | 1.18 | 2 (2 to 4) | 3.24 (2.9 to 3.57) | 1.42 | -1.18 | 0.32 |
| M8 | O | 346 | 3 | 1.13 | 2 (2 to 4) | 2.74 (2.62 to 2.86) | 1.29 | -0.88 | 0.12 |
| ✮ M8 | P | 224 | 2 | 1.12 | 1 (2 to 3) | 2.55 (2.41 to 2.7) | 1.25 | -0.86 | 0.23 |
| ✮ M8 | N | 52 | 3 | 1.05 | 1 (3 to 4) | 3.33 (3.03 to 3.62) | 1.13 | -0.67 | -0.18 |
| ◈ M9 | O | 335 | 4 | 0.7 | 1 (4 to 5) | 4.3 (4.22 to 4.37) | 0.49 | 0.6 | -0.8 |
| M9 | P | 223 | 4 | 0.67 | 1 (4 to 5) | 4.35 (4.27 to 4.44) | 0.46 | 0.61 | -0.83 |
| M9 | N | 52 | 4 | 0.59 | 1 (4 to 5) | 4.27 (4.1 to 4.44) | 0.36 | -0.55 | -0.16 |
| ◈ M10 | O | 333 | 4 | 0.72 | 1 (4 to 5) | 4.17 (4.09 to 4.25) | 0.52 | 0.7 | -0.66 |
| M10 | P | 224 | 4 | 0.69 | 1 (4 to 5) | 4.25 (4.15 to 4.34) | 0.48 | -0.21 | -0.53 |
| M10 | N | 49 | 4 | 0.77 | 1 (4 to 5) | 4.06 (3.84 to 4.28) | 0.6 | 3.3 | -1.19 |
| ◈ M11 | O | 331 | 4 | 0.92 | 1 (3 to 4) | 3.67 (3.57 to 3.77) | 0.86 | 0.07 | -0.44 |
| M11 | P | 224 | 4 | 0.94 | 1 (3 to 4) | 3.68 (3.56 to 3.81) | 0.88 | -0.13 | -0.41 |
| M11 | N | 48 | 4 | 0.73 | 1 (3 to 4) | 3.79 (3.58 to 4.01) | 0.55 | -1.09 | 0.35 |
| M12 | O | 329 | 3 | 1.28 | 2 (2 to 4) | 2.91 (2.77 to 3.05) | 1.65 | -1.09 | 0.1 |
| M12 | P | 221 | 3 | 1.31 | 2 (2 to 4) | 3 (2.83 to 3.17) | 1.73 | -1.17 | 0.05 |
| M12 | N | 50 | 3 | 1.16 | 2 (2 to 4) | 2.74 (2.41 to 3.07) | 1.38 | -0.9 | 0.06 |
| ◈ M13 | O | 334 | 4 | 1.15 | 1.75 (3 to 4.75) | 3.64 (3.51 to 3.76) | 1.33 | -0.32 | -0.67 |
| M13 | P | 223 | 4 | 1.16 | 2 (3 to 5) | 3.69 (3.54 to 3.84) | 1.34 | -0.41 | -0.63 |
| M13 | N | 51 | 4 | 1.13 | 1 (3 to 4) | 3.55 (3.23 to 3.87) | 1.29 | -0.06 | -0.86 |
| ◈ M14 | O | 334 | 4 | 0.81 | 1 (4 to 5) | 4.25 (4.16 to 4.33) | 0.65 | 2.09 | -1.26 |
| M14 | P | 225 | 4 | 0.73 | 1 (4 to 5) | 4.32 (4.23 to 4.42) | 0.53 | 0.95 | -1 |
| M14 | N | 51 | 4 | 1.09 | 1 (4 to 5) | 3.94 (3.63 to 4.25) | 1.22 | 1.03 | -1.24 |
| ◈ M15 | O | 334 | 4 | 0.68 | 1 (4 to 5) | 4.38 (4.31 to 4.46) | 0.46 | 1.13 | -0.99 |
| M15 | P | 224 | 4 | 0.64 | 1 (4 to 5) | 4.42 (4.33 to 4.5) | 0.42 | 0.58 | -0.86 |
| M15 | N | 50 | 5 | 0.75 | 1 (4 to 5) | 4.4 (4.19 to 4.61) | 0.57 | 1.97 | -1.37 |
| ◈ M16 | O | 335 | 4 | 0.71 | 1 (4 to 5) | 4.36 (4.29 to 4.44) | 0.51 | 0.89 | -1.01 |
| M16 | P | 224 | 5 | 0.72 | 1 (4 to 5) | 4.39 (4.3 to 4.49) | 0.53 | 1.34 | -1.18 |
| M16 | N | 51 | 5 | 0.61 | 1 (4 to 5) | 4.47 (4.3 to 4.64) | 0.37 | -0.5 | -0.68 |
| Res = Responses; Pop = Population (O = overall, P = physicians, N = nurses); N = Number of observations; x͂ = Median; SD = Standard Deviation; IQR = Interquartile Range; 95% CI = 95% Confidence Interval; Var = Variance; Kurt = Kurtosis; Skew = Skewness. ^a^ Responses marked with diamonds (◈) have a median ≥ 4; Responses marked with stars (✮) indicate statistically significant differences between professional groups. | | | | | | | | | |

Survey Items M17-M18.

| Res^a^ | Pop^b^ | N | x͂ | SD | IQR | 95% CI | Var | Kurt | Skew |
| --- | --- | --- | --- | --- | --- | --- | --- | --- | --- |
| ◈ M17 | O | 333 | 5 | 0.64 | 1 (4 to 5) | 4.46 (4.39 to 4.53) | 0.41 | 1.23 | -1.03 |
| M17 | P | 223 | 5 | 0.63 | 1 (4 to 5) | 4.48 (4.4 to 4.56) | 0.4 | 0.94 | -1.03 |
| M17 | N | 52 | 4.5 | 0.5 | 1 (4 to 5) | 4.5 (4.36 to 4.64) | 0.25 | -2 | 0 |
| ◈ M18 | O | 330 | 4 | 1.03 | 2 (3 to 5) | 3.79 (3.68 to 3.9) | 1.07 | -0.14 | -0.72 |
| M18 | P | 221 | 4 | 1.04 | 2 (3 to 5) | 3.77 (3.63 to 3.91) | 1.08 | -0.26 | -0.65 |
| M18 | N | 50 | 4 | 0.93 | 1 (4 to 5) | 4.02 (3.75 to 4.29) | 0.88 | 0.19 | -0.94 |
| Res = Responses; Pop = Population (O = overall, P = physicians, N = nurses); N = Number of observations; x͂ = Median; SD = Standard Deviation; IQR = Interquartile Range; 95% CI = 95% Confidence Interval; Var = Variance; Kurt = Kurtosis; Skew = Skewness. ^a^ Responses marked with diamonds (◈) have a median ≥ 4; Responses marked with stars (✮) indicate statistically significant differences between professional groups. | | | | | | | | | |

Item-level Mann-Whitney U Test Results with Benjamini-Hochberg Correction by Professional Group Across All Items.

| Item | *U* | *p* | *p_adj* | Reject H_0_ |
| --- | --- | --- | --- | --- |
| D7 | 3314 | 5.20526E-07 | 2.29031E-05 | Yes |
| M8 | 3665.5 | 1.78706E-05 | 0.000393153 | Yes |
| M3 | 7519 | 4.22377E-05 | 0.000619487 | Yes |
| D8 | 4089.5 | 0.000703151 | 0.00667892 | Yes |
| S4 | 3608.5 | 0.000758968 | 0.00667892 | Yes |
| S10 | 4325.5 | 0.003614819 | 0.024186347 | Yes |
| S13 | 4368 | 0.003847828 | 0.024186347 | Yes |
| D9 | 4513.5 | 0.013256191 | 0.069193115 | No |
| M6 | 6820.5 | 0.014153137 | 0.069193115 | No |
| D2 | 6807 | 0.025615415 | 0.110963055 | No |
| M14 | 6756.5 | 0.029921576 | 0.110963055 | No |
| M5 | 6553.5 | 0.030262651 | 0.110963055 | No |
| M1 | 5044.5 | 0.0566052 | 0.187442896 | No |
| S3 | 5665 | 0.062708295 | 0.187442896 | No |
| S14 | 6612 | 0.063900987 | 0.187442896 | No |
| S15 | 4796 | 0.073661452 | 0.202568994 | No |
| M18 | 4764 | 0.108614238 | 0.281119203 | No |
| M10 | 6176.5 | 0.130035319 | 0.316463139 | No |
| D5 | 4864 | 0.136654537 | 0.316463139 | No |
| S1 | 5127.5 | 0.178373282 | 0.39242122 | No |
| M2 | 5912.5 | 0.19786533 | 0.414120715 | No |
| S2 | 4284.5 | 0.209268401 | 0.414120715 | No |
| S11 | 4926.5 | 0.225633958 | 0.414120715 | No |
| M12 | 6117.5 | 0.225884026 | 0.414120715 | No |
| M9 | 6332 | 0.249714567 | 0.439497638 | No |
| D11 | 4837 | 0.282977988 | 0.478885826 | No |
| D6 | 5217 | 0.298295164 | 0.486110638 | No |
| M13 | 6082 | 0.421080341 | 0.652723098 | No |
| S12 | 5387.5 | 0.43020386 | 0.652723098 | No |
| S9 | 6023.5 | 0.474553949 | 0.696012458 | No |
| S6 | 5370 | 0.583253755 | 0.782724053 | No |
| D3 | 5462 | 0.608270904 | 0.782724053 | No |
| S8 | 4923.5 | 0.611919671 | 0.782724053 | No |
| S7 | 5541 | 0.616228137 | 0.782724053 | No |
| S5 | 5656 | 0.622621406 | 0.782724053 | No |
| M16 | 5511 | 0.661725195 | 0.793637325 | No |
| M11 | 5174.5 | 0.667376841 | 0.793637325 | No |
| M7 | 5821.5 | 0.746257231 | 0.86408732 | No |
| M17 | 5902 | 0.819668284 | 0.901262336 | No |
| D10 | 5722.5 | 0.837387296 | 0.901262336 | No |
| D4 | 5803 | 0.844610321 | 0.901262336 | No |
| M15 | 5520 | 0.860295866 | 0.901262336 | No |
| D1 | 5726 | 0.927791011 | 0.93987009 | No |
| M4 | 5549.5 | 0.93987009 | 0.93987009 | No |
